# Cooperation-based sperm clusters mediate sperm oviduct entry and fertilization

**DOI:** 10.1007/s13238-021-00825-y

**Published:** 2021-03-01

**Authors:** Yongcun Qu, Qi Chen, Shanshan Guo, Chiyuan Ma, Yonggang Lu, Junchao Shi, Shichao Liu, Tong Zhou, Taichi Noda, Jingjing Qian, Liwen Zhang, Xili Zhu, Xiaohua Lei, Yujing Cao, Wei Li, Wei Li, Nicolas Plachta, Martin M. Matzuk, Masahito Ikawa, Enkui Duan, Ying Zhang, Hongmei Wang

**Affiliations:** 1 State Key Laboratory of Stem Cell and Reproductive Biology, Institute of Zoology, Chinese Academy of Sciences, Beijing, 100101 China; 2 University of Chinese Academy of Sciences, Beijing, 100049 China; 3 Division of Biomedical Sciences, School of Medicine, University of California, Riverside, CA 92521 USA; 4 Research Institute for Microbial Diseases, Osaka University, Suita, Osaka Japan; 5 Department of Physiology and Cell Biology, Reno School of Medicine, University of Nevada, Reno, NV USA; 6 Institute of Molecular and Cell Biology, A*STAR, Singapore, Singapore; 7 Center for Drug Discovery, Department of Pathology and Immunology, Baylor College of Medicine, Houston, TX 77030 USA; 8 Division of Biology and Biological Engineering, California Institute of Technology, Pasadena, CA USA; 9 Institute for Stem Cell and Regeneration, Chinese Academy of Sciences, Beijing, 100101 China; 10 The Key Laboratory of Cell Proliferation and Regulation Biology, Ministry of Education, Institute of Cell Biology, Beijing Normal University, Beijing, 100875 China

**Dear Editor,**

Sperm cooperation has been observed in multiple species (Pizzari and Foster, 2008), yet its existence and benefit for reproductive success in mammals remains underexplored. Here, combining tissue-clearing with deep three-dimensional (3D) imaging, we demonstrate that postcopulatory mouse sperm congregate into unidirectional sperm cooperative clusters at the utero-tubal junction (UTJ), a key physical barrier for passage into the oviduct. Reducing sperm number in male mice by unilateral vasoligation (Uni-Vas) or busulfan-treatment impairs sperm cluster formation and oviduct entry. Interestingly, *Tex101*^−/−^ male mouse produce normal sperm number, motility and morphology, yet their sperm cannot form clusters and fail to pass through the UTJ, which is at least in part due to the altered tail beating pattern of the *Tex101* null sperm. Moreover, *Tex101* null sperm’s defect in oviduct entry cannot be rescued by the presence of wild-type (WT) sperm in the same uteri by sequential mating, suggesting sperm cooperative cluster as an essential behavior contributing to male fertility.

Sperm must overcome significant obstacles within the female reproductive track to reach and fertilize the egg. In many mammalian species, the UTJ is a narrow tube with multiple crypts and viscous fluid. This structure, connects the uterus to the oviduct and functions as an efficient physical barrier that blocks most sperm in the uterus, allowing only a few to enter the oviduct (Holt and Fazeli, 2016) (Fig. 1C). Although this phenomenon has been well-documented for a long time, the mechanisms underlying how sperm pass through the UTJ remain largely unknown.

**Figure 1:**
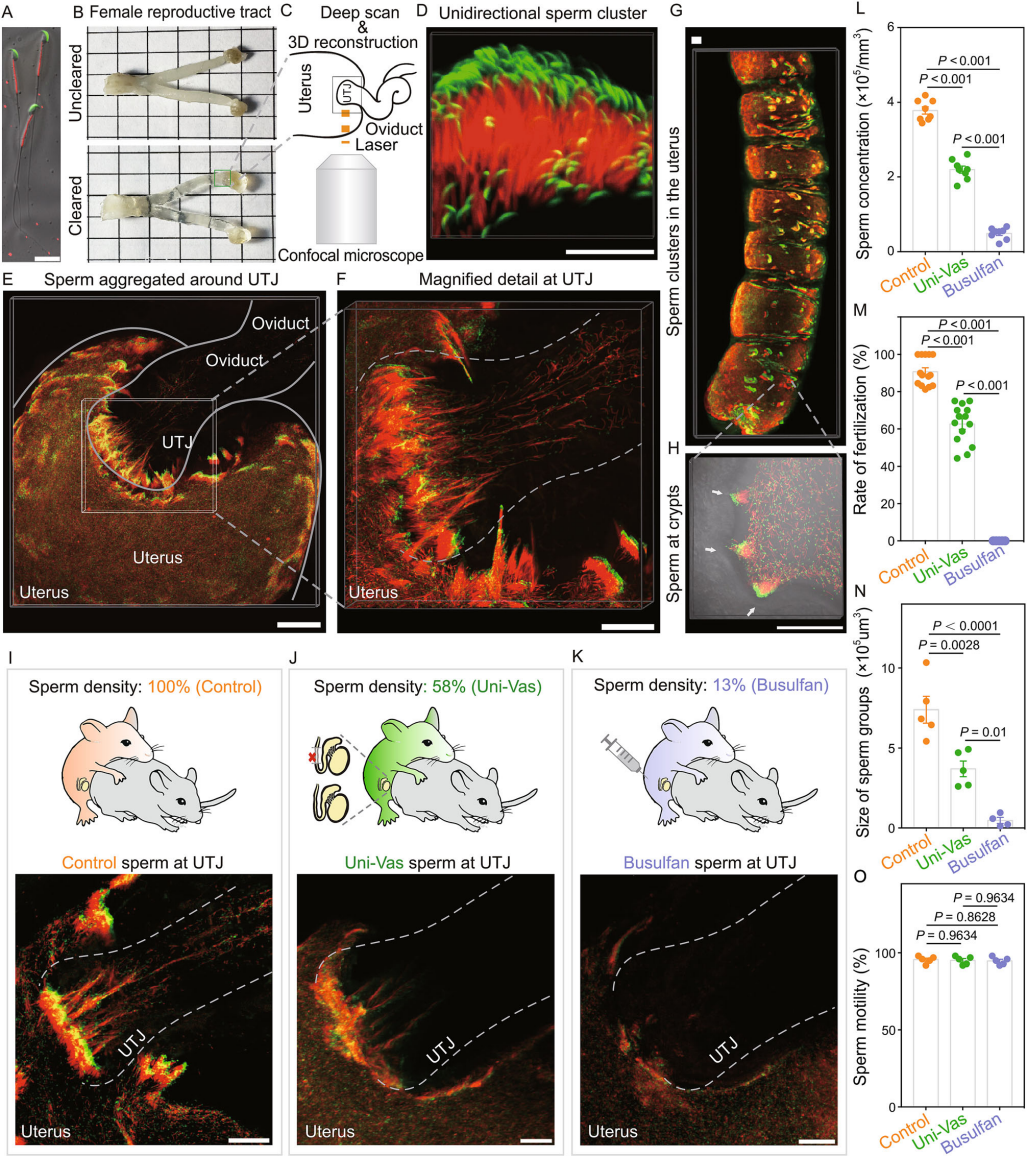
**Sperm clusters at the UTJ and in the uterus one hour after coitus, regulated by sperm number**. (A) Sperm with GFP-labeled acrosome and RFP-labeled mitochondria (CAG/su9-DsRed2, Acr3-EGFP). Scale bar, 20 μm. (B) Female reproductive tract before and after clearing. (C) Schematic of 3D imaging. (D) Sperm cluster with sperm head oriented in the same direction. Scale bar, 30 μm (E) 3D imaging of sperm aggregated around the UTJ. Scale bar, 200 μm. (F) Magnified detail of sperm cluster at the UTJ. Scale bar, 200 μm. (G) 3D imaging of sperm clusters in the uterus. Scale bar, 200 μm. (H) Sperm cluster distribution in uterine crypts (white arrow). Scale bar, 200 μm. (I–K) Schematic diagrams of mating procedure (top) and 3D visualization of sperm behavior at the UTJ (bottom). Scale bars, 100 μm. (L) Analysis of Control, Uni-Vas and Busulfan-treated male sperm concentration in the uteri. *n* = 8 different visual fields from 4 male mice each. (M) *In vivo* fertilization rate. *n* = (14 control male mice, 14 Uni-Vas male mice and 5 Busulfan-treated male mice). (N) Quantification of the size of sperm groups (volumetric analysis of the sperm fluorescent signal) in control, Uni-Vas and Busulfan-treated male at the UTJ (two hours after coitus). *n* = (5 control male mice, 5 Uni-Vas male mice and 4 Busulfan-treated male mice). (O) Sperm motility of Control, Uni-Vas and Busulfan-treated male mice. *n* = 5 male mice each. The results are shown as mean ± SEM

It has been previously suggested that linear progressive motility can increase the efficiency of sperm to pass through the UTJ . However, emerging evidence over past decades indicated that sperm motility alone is not sufficient for sperm oviduct entry (Holt and Fazeli, 2016; Fujihara et al., 2018), as demonstrated by many mouse knockout strains showing normal sperm motility but defective oviduct entry (Fujihara et al., 2018), suggesting additional mechanisms. A recent study in mouse suggested that sperm behaviors could be critical for sperm moving through the female reproductive tract (Wang and Larina, 2018).

Interestingly, increasing evidence from molluscs to mammals have shown that sperm can group together *in vitro*, a collective behavior which has been termed sperm cooperation, and might be beneficial for mutual advantages to improve fertilization probability (Pizzari and Foster, 2008). For example, several rodent sperm were found to form aggregates in the culture medium to increase progressive motility and to prevent premature acrosome reaction (Pizzari and Foster, 2008). Yet, it remains unclear whether and how mammalian sperm display such behavior *in vivo*, mainly due to the high opacity and inaccessibility of female reproductive tract.

To address this issue, we employed whole-organ clearing technologies, optimized from previous work (Yang et al., 2014), and this approach rendered the female reproductive tract with high transparency (Fig. 1B). To visualize the intrauterine sperm behaviors at one- and two-hour postcoitum, we used transgenic sperm fluorescently labeled with a red (DsRed2) midpiece, and a green (GFP) acrosome (Hasuwa et al., 2010) (Fig. 1A). Combining with 3D confocal imaging and reconstruction (Fig. 1C), we found that sperm densely assembled into clusters at the UTJ, with sperm heads and tails spatially oriented in the same direction facing the oviduct (Figs. 1D–F, S1 and Movie S1), and there are dozens to hundreds of sperm in the sperm cluster. In addition to the sperm clusters found at the UTJ, we found similar clusters inside the uterus, in regions where crypts form (Fig. 1G and 1H). These results suggest that sperm clusters may not “intentionally” aggregate at the UTJ, but rather they scout the whole uterus and accumulate at all crypt-like regions, including the UTJ.

The observation of sperm clusters at the UTJ raised the question whether this population-based sperm behavior is related to sperm oviduct entry. If true, the sperm entry into the oviduct should be hampered when the size of the sperm clusters is reduced. To test this hypothesis, we first generated a Uni-Vas male mice model to approximately reduce the sperm number in half (Fig. 1J). Indeed, after two weeks’ recovery, the sperm concentration significantly decreased to 58.08% of control level (Fig. 1L). Using the same method to generate the 3D reconstructed female reproductive tract with sperm, we found that the Uni-Vas males showed a significant decrease in the size of sperm clusters (volumetric analysis of sperm fluorescent signal) at the UTJ, compared to control mice (Fig. 1I, 1J and 1N). Furthermore, the *in vivo* fertilization rate by Uni-Vas males was markedly reduced to 62.62% (control mice: 90.78%) (Fig. 1M). These findings demonstrate that sperm number is a contributing factor to the size of sperm clusters at the UTJ and is highly related to the number of sperm that can enter into the oviduct.

To further reduce sperm number, we used another male mouse model by injecting a single dose of busulfan (Fig. 1K), a cytotoxic regent that can kill spermatogonia and induce oligospermia or azoospermia. Two months after a single low-dose (17 mg/kg) busulfan treatment, the sperm concentration reduced to 5.65%–17.9% (from 5 mice, highest: 17.90% and lowest: 5.65%) of control level (Fig. 1L), causing complete infertility in these mice (Fig. 1M), although the motility of the remaining sperm showed no difference compared with control mice (Fig. 1O). Importantly, 3D imaging results showed that despite the fact that the sperm number decreased substantially, some sperm could still reach the UTJ; but in this situation, little or no sperm clusters could form at the UTJ (Fig. 1K and 1N) and no sperm could enter the oviduct (Fig. 1M). These results further supported the conclusion that a sufficient number of sperm is essential for the efficient formation of sperm clusters at the UTJ, which is highly related to the ability of sperm to pass through the UTJ and may explain the observation that the inseminated sperm number was associated with reproductive efficiency in individuals. When the sperm number decrease below a certain threshold, the sperm cannot pass through the UTJ. In addition, according to our mating data, we found that when sperm concentration was close to 25% of pretreatment level, sperm could be found in the oviduct and the mice showed subfertility (~22% fertility rate compared to the control mice). Thus, we deduced that the minimal sperm counts that can support the sperm entrance into the oviduct could be between 17.9% to 25% of control (Minimal number needed for successful penetration at the UTJ may be between 160–480, which was a rough calculated according to our available data). This finding is consistent with previous evidence suggesting that ~20% of normal sperm counts is a threshold for male mice fecundity.

In addition to the importance of the sperm counts for sperm clustering and fertility, other factors should be involved. Indeed, recent studies have reported that over ten different knockout mouse models that showing normal sperm counts, motility, and morphology, but the sperm from these mutant mice cannot migrate through the UTJ and these male mice are infertile (Fujihara et al., 2018). Could these defects relate to sperm clustering? To test this hypothesis, we used one of these knockout strains, *Tex101*^−/−^ (Li et al., 2013) to test the sperm ability to aggregate *in vitr*o and to form clusters at the UTJ *in vivo*.

For the *in vitro* aggregation experiment, cauda epididymal sperm of WT and *Tex101*^−/−^ mice were separately released into the M16 medium and then the numbers of the sperm aggregation (>10 sperm per aggregation) between these two groups were compared. As shown in Fig. S2, the aggregation ability of *Tex101* null sperm was severely damaged. Similar defects of *in vitro* aggregation have also been observed in other gene knockout mice that show normal sperm counts but are infertile (Han et al., 2010).

To further examine whether the *Tex101* null sperm can form sperm clusters *in vivo*, we crossed the *Tex101*^−/−^ mice into the same transgenic mice (red (DsRed2) midpiece, and green (GFP) acrosome), mated these mutant males with normal females, and then used the same tissue-clearing and 3D imaging analyses as we did for WT sperm. As shown in Fig. 2, the 3D imaging showed that the sperm of *Tex101*^−/−^ mice were unable to form clusters *in vivo* (Figs. 2A, 2B, 2E, 2F and S3); instead, they distributed irregularly near the UTJ (Fig. 2C), and most of them were blocked outside or at the entry of the UTJ (Fig. 2B and Movie S2).

**Figure 2:**
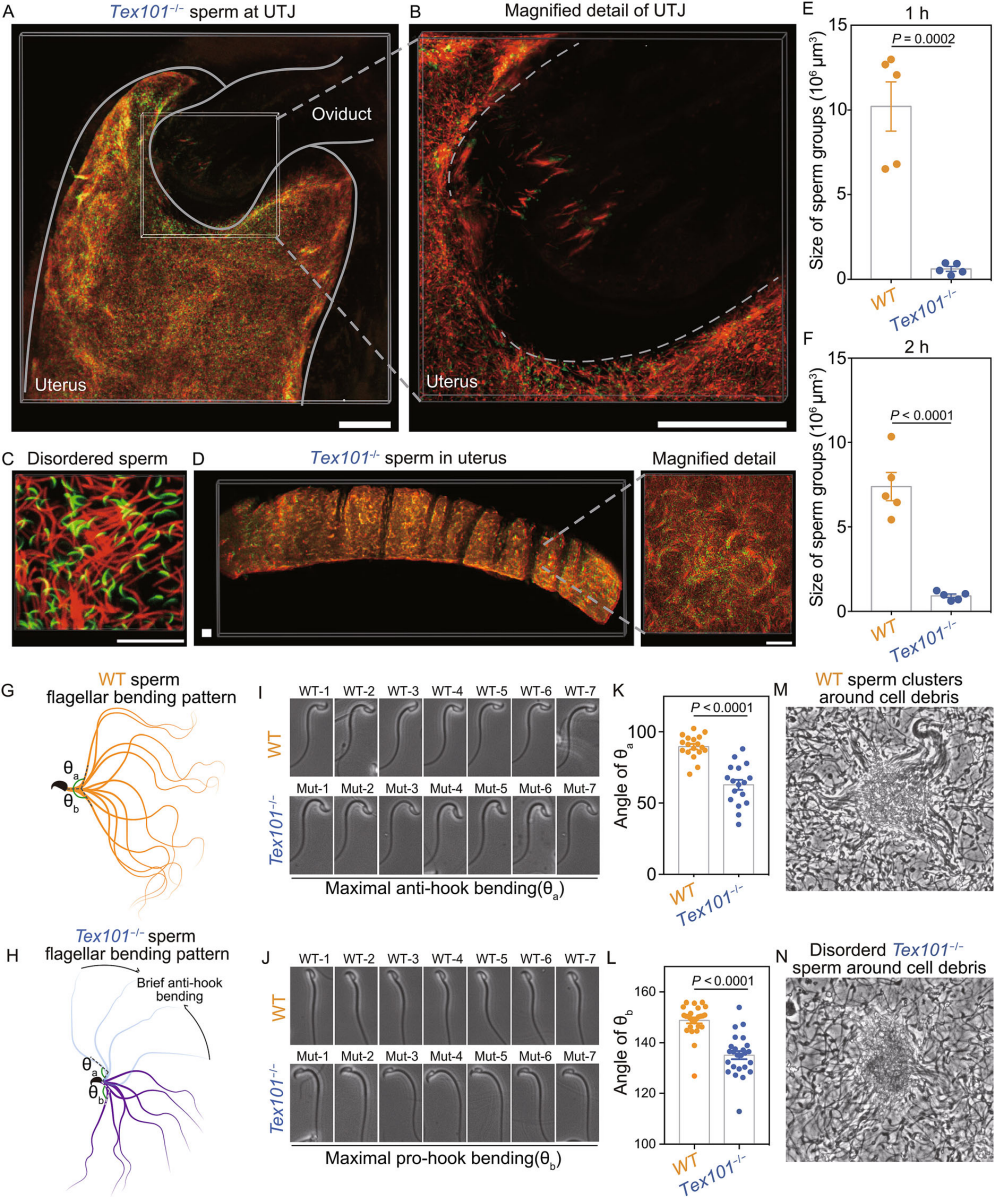
**Sperm cluster mediate sperm oviductal entry and its formation associated with sperm tail bending pattern**. (A) *Tex101* null sperm fail to form clusters at the UTJ. Scale bar, 200 μm. (B) Magnified detail of *Tex101* null sperm at UTJ. Scale bar, 200 μm. (C) *Tex101* null sperm displaying disordered distribution. Scale bar, 30 μm. (D) *Tex101* null sperm remain scattered in the uterus. Scale bars, 200 μm. (E and F) Quantification of the size of sperm groups (volumetric analysis of the sperm fluorescent signal) from WT and *Tex101*^−/−^ mice at the UTJ at one (E) and two hours (F) after coitus on a red fluorescence signal-generating surface. Results are shown as the mean ± SEM, *n* = 5 male mice each in (E) and (F). (G) WT sperm flagellar bending pattern. (H) *Tex101* null sperm flagellar bending pattern. (I) Images of WT (top) and *Tex101* null (bottom) sperm maximal anti-hook bending. (J) Images of WT (top) and *Tex101* null (bottom) sperm maximal pro-hook bending. (K) Angle of WT and *Tex101* null sperm maximal anti-hook bending around the neck. (L) Angle of WT and *Tex101*^−/−^ sperm maximal pro-hook bending around the neck. The results are shown as the mean ± SEM, *n* = 4 male mice each. (M) WT sperm behavior around cell debris. (N) *Tex101* null sperm behavior around cell debris

Moreover, according to the volumetric analysis of the sperm groups (depending on the fluorescent signal) in the uterus, we also found that *Tex101* null sperm could not form large clusters within the uterus, but only have tiny groups with disordered orientation compared with the WT mice (Figs. 2D, and S4). These results support the idea that in addition to sperm counts, morphology and motility, the sperm clustering behavior could be another essential contributing factor for efficient sperm migration and fertility.

Subsequently, we tested whether those sperm that cannot form sperm cluster, such as those derived from the *Tex101*^−/−^ mice, would be expected to enter the oviduct with the help of normal sperm clusters. To test this, we developed a mating procedure allowing a wild-type female to sequentially mate with two transgenic males bearing sperm with different markers. This sequential mating experiment is possible as mice are promiscuous. We used WT (GFP-labeled sperm tail) (Wang et al., 2016) followed by *Tex101*^−/−^ (RFP-labeled sperm tail) mice (Hasuwa et al., 2010), and *Tex101*^−/−^ (RFP-labeled sperm tail) followed by WT (GFP-labeled sperm tail) mice (Fig. S5A and S5B). Video monitoring confirmed that successful mating with the second male occurred as early as 45 min after the first male. Imaging of WT and *Tex101* null sperm in the uterine horns after sequential mating revealed that only WT sperm formed clusters and entered the oviduct. By contrast, *Tex101* null sperm could not migrate into the oviduct, albeit they entered the uterus before the WT sperm (Fig. S5C and S5D). The imaging data were further supported by functional results, showing that after sequential mating, all offspring derived from WT and none from *Tex101* null sperm, independently of mating order (Table 1). Therefore, although the sperm number in the uterus increased substantially after sequential mating, the scattered *Tex101* null sperm cannot either form clusters or take advantage of the sperm clusters of WT sperm to enter the oviduct, indicating the sperm clustering itself is an intrinsic property responsible for sperm entry into the oviduct.

**Table 1:** **Litter size and genotype after sequential mating**

		Number of pups		
Mating order	Number of cages	Litter size	WT	*Tex101*^-/-^
WT first *Tex101*^-/-^ second	1	19	19/19	0/19
	2	9	9/9	0/9
	3	11	11/11	0/11
	4	5	5/5	0/5
	5	13	13/13	0/13
	6	12	12/12	0/12
	7	6	6/6	0/6
	8	9	9/9	0/9
*Tex101*^-/-^ first WT second	1	12	12/12	0/12
	2	14	14/14	0/14
	3	7	7/7	0/7
	4	8	8/8	0/8
	5	9	9/9	0/9
	6	5	5/5	0/5
	7	10	10/10	0/10
	8	11	11/11	0/11

Why do the *Tex101* null sperm not cluster? One possibility is that this might be caused by a lack of “sticky” molecules as we previously discussed (Li et al., 2013). In the current study, we further explored an alternative explanation that the lack of grouping behavior might be related to abnormal sperm swimming pattern and altered hydrodynamics, as there is a recent study showing that sperm collective behavior formation is influenced by sperm waveform dynamics (Ishimoto and Gaffney, 2018). To test this hypothesis, we released sperm (WT vs. *Tex101* null) from uteri 1.5 h after coitus (mating with normal C57 female) and put the sperm into the viscoelastic medium, followed by detailed video recording/analysis of sperm swimming in a frame-by-frame manner. This detailed analysis has led to an exciting discovery that the WT and *Tex101* null sperm indeed showed different swimming patterns. As shown in Fig. 2G and Movie S3, the sperm flagellar bending pattern of anti-hook and pro-hook were almost symmetric distributed in WT sperm. However, the *Tex101* null sperm have two asymmetric flagellar bending patterns, showing a prolonged pro-hook bending and a brief anti-hook bending besides the pro-hook bending (Fig. 2H and Movie S4); the extent of both pro-hook bending and a brief anti-hook (reflected by the minimal value of angle θ in Fig. 2G and 2H) in *Tex101* null sperm were more dramatic than the WT sperm (Fig. 2I–L). This different swimming pattern might hold the key to explain why *Tex101* null sperm cannot form clusters, because this more dramatic asymmetric anti-hook/pro-hook swimming pattern might make the sperm head difficult to “attach” to the objectives. This may partially explain why the *Tex101* null sperm cannot group at the uterine crypts or the UTJ.

Moreover, during the detailed recording on the sperm that are freshly released from the uteri (1.5 h after coitus), we have obtained further insight on how the sperm might change their moving pattern after they form cluster. In Fig. 2M and Movie S5, we found that when a cluster of sperm swimming around cell debris, all the sperm tails wave in a synchronized manner, generating a strong tail pendulum force pushing the cell/debris. Since the sperm clusters at the UTJ were positioned in a unified direction towards the UTJ (Fig. 1D–F), this very force generated by the synchronized sperm tail beating might transiently push the UTJ open and allow the sperm at the center of the entrance to penetrate into the oviduct. Whereas the *Tex101* null sperm could not form the clusters around cell/debris and form this unified force (Fig. 2N and Movie S6). The data obtained from *ex vivo* sperm videos could provide explanations on two facts: 1) a reduced sperm number, either in the Uni-vas or busalfan treatment experiments (Fig. 1I–O), decreases the overall force generated by the synchronized sperm tail beating, and when the generated force is below a certain threshold it cannot efficiently push the UTJ open; 2) The *Tex101* null sperm never achieve the threshold of force to push open the UTJ because it cannot form sperm clusters at the UTJ (Fig. 2A and 2B).

In the present work, we demonstrate that mouse sperm establish unidirectional sperm clusters at the UTJ, and that this intrauterine sperm cooperation-based behavior contribute to sperm oviduct entry and fertility. Although *in vitro* experiments had revealed that mouse sperm aggregation might be related to male fertility (Han et al., 2010), it remains an unsolved issue whether this sperm cooperation behavior exist within the female tract after coitus. Traditional histological methods applied on thin tissue sections provide limited spatial information about sperm behavior *in vivo*. We addressed this technical challenge by combining optical sectioning microscopy and optimized tissue clearing techniques . As the post-copulation uterus contains liquid semen, we used hydrogel-based tissue clearing (Yang et al., 2014) to render the post-copulation female reproductive tract with copulation plug transparent. This approach improved semen fluid density and turned uterine contents into an elastic gel, which enabled the fixation of large sperm population and the preservation their position as they were *in vivo*. Using this method, we successfully discovered the sperm cooperation behavior in the female reproductive tract after mating.

Why do sperm aggregate to form groups when they swim through the female reproductive tract? From an evolutionary perspective, sperm evolution is mainly driven by two forces: postcopulatory sexual selection from female cryptic choice and sperm competition environment between males (Firman et al., 2017). From the female perspective, making all her eggs fertilized by health sperm is the priority. Thus the female reproductive tract designs complex barriers to select sperm (Holt and Fazeli, 2016). But from the male part, all the sperm are potentially valuable and could be in some way to facilitate fertilization to overcome the female “obstacles” and to engage with competitor’s sperm in a polyandrous environment. In this situation, males may evolve sperm cooperation behavior to efficiently transport through the female tract and response to dramatic sperm competition environment (Pizzari and Foster, 2008). Experimental studies have found that the coitus induces a viscous oviduct fluid flow towards the UTJ, which is thought to reduce sperm beat frequency and hinder their migration (Miki and Clapham, 2013). Our current study has provided further compelling evidence that sperm clustering *in vivo* is functionally important as the sperm clusters could provide enhanced force to push the UTJ open, thus enable the sperm entrance into the oviduct. When the sperm cannot form cluster, such as in the case of *Tex101*^−/−^ mice, the sperm are blocked outside the narrow UTJ. Interestingly, we also found that sperm predominately aggregate into clusters in uterine crypts, which are an anatomical structure similar to the UTJ. Although lacking of exact evidence, we speculated that sperm might find their way to the UTJ via a trial-and-error process, and the mechanism of sperm cluster formation may also involve sperm-female and sperm-sperm communication .

The molecular and physiological mechanisms by which sperm congregate into the sperm cluster within the intrauterine environment remain unknown. Plausibly, they may rely on the sticky nature of sperm, which is lost in some gene knockout mice (Han et al., 2010), for example, *Tex101*^−/−^ mutants. To date, more than 10 factors have been reported to be important for sperm UTJ migration and most of them are related to ADAM3 maturation. ADAM3 has been proposed as the potential key sticky molecular for proper sperm function, yet how it is causally related to the sperm migration through the UTJ remains unclear (Fujihara et al., 2018). Additionally, we found that the sperm movement pattern may also contribute to the sperm cluster formation (Fig. 2), which resonates with a previous report that collective behavior could be driven by hydrodynamic interactions regulated by sperm waveform dynamics (Ishimoto and Gaffney, 2018). Following ejaculation, sperm are in close proximity with each other at a high concentration, which could facilitate sperm-sperm interactions and generate synchronized tail beating to enhance the sperm force to push objects to which they attached (e.g., the UTJ). Furthermore, the highly viscous fluid within the female reproductive tract with low Reynolds number (Yang et al., 2008), may hydrodynamically promote rapid sperm clustering and synchronized swimming, whereas decreased sperm density and abnormal beating wavelength may disrupt sperm clustering (Ishimoto and Gaffney, 2018). Moreover, the confined geometry of the convoluted epithelial lining may also contribute to sperm clustering, as most clusters aggregate within uterine crypts and UTJ. Previous studies have suggested sperm has strong corner-swimming behavior, which originates from hydrodynamic interaction of sperm with surface of the corner (Nosrati et al., 2016). Notably, similar population-based sperm behavior may also exist in human, and it has been reported that high density of sperm was located at crypts of human cervix (Insler et al., 1980), where the sperm may similarly form clusters and generated synchronized force to facilitate their moving into uterus.

Another important finding of our work is that decreased sperm number hampered sperm cluster formation at the UTJ and sperm oviduct entry. According to our data, despite having normal morphology and motility, when sperm counts drop to 17.9% (or less) of control level, sperm clusters are hard to form and none of the sperm can enter the oviduct.

Finally, our study is also highly related to human fertility. While sperm counts, motility, and morphology are clinically recognized as indicators of male fecundity, it has been suggested that these factors often cannot fully account for or predict clinical diagnosis. Defects in sperm clustering may account for certain infertility or subfertility problems in humans with normal sperm counts and morphology. Our data may also explain subfertility in oligospermia (low sperm counts), which could be due to insufficient sperm clustering in the female reproductive tract and failure to migrate through major barriers such as the cervix. In conclusion, our study revealed that sperm cooperation behavior takes part in the process of fertilization and is linked to the male fertility, which would provide a new insight for the research in evolutionary and reproductive biology.

## Supplementary Information

Below is the link to the electronic supplementary material.

Supplementary material 1 (PDF 856 kb)

Supplementary material 2 (MOV 2787 kb)

Supplementary material 3 (MOV 2760 kb)

Supplementary material 4 (MOV 9064 kb)

Supplementary material 5 (MOV 9116 kb)

Supplementary material 6 (MOV 4666 kb)

Supplementary material 7 (MOV 4671 kb)
