# Supplementary material for: Cooperation-based sperm clusters mediate sperm oviduct entry and fertilization
Source: Protein Cell. 2021 Mar 1;12(10):810–7. doi: 10.1007/s13238-021-00825-y (PMC8464547; doi:10.1007/s13238-021-00825-y)
Supplement: Supplementary file 1 — Supplementary material 1 (PDF 856 kb) [file 13238_2021_825_MOESM1_ESM.pdf]

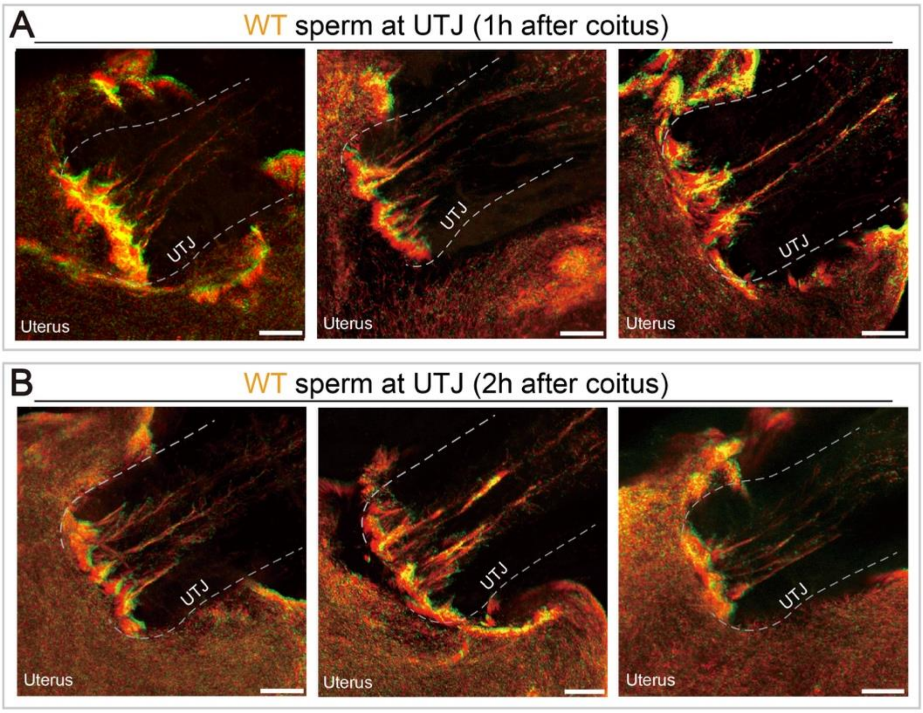

Figure S1. WT sperm behavior at UTJ. (A) WT sperm behavior at UTJ 1h after coitus. (B) WT sperm behavior at UTJ 2h after coitus. scale bars, 200μm.

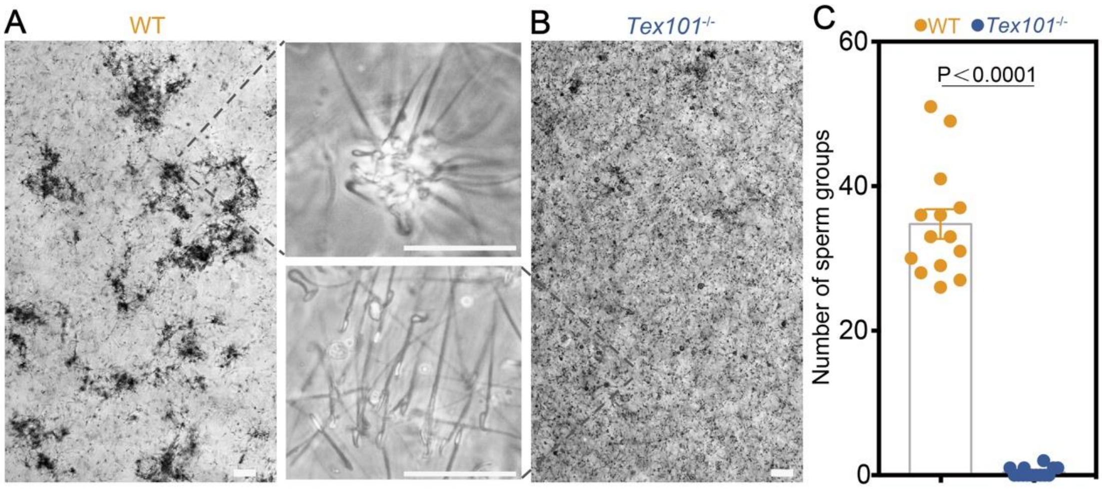

Figure S2. Comparison of WT and *Tex101*$^{-/-}$ aggregation ability *in vitro.* (A) WT sperm aggregate during *in vitro* culture. (B) *Tex101*$^{-/-}$ sperm show impaired aggregation. (C) Quantification of aggregated sperm groups (> 10 sperm per group) for WT and *Tex101*$^{-/-}$ mice. The results are shown as the mean ± SEM, n = 14 different visual fields from 4 male mice each. Scale bars, 50μm.

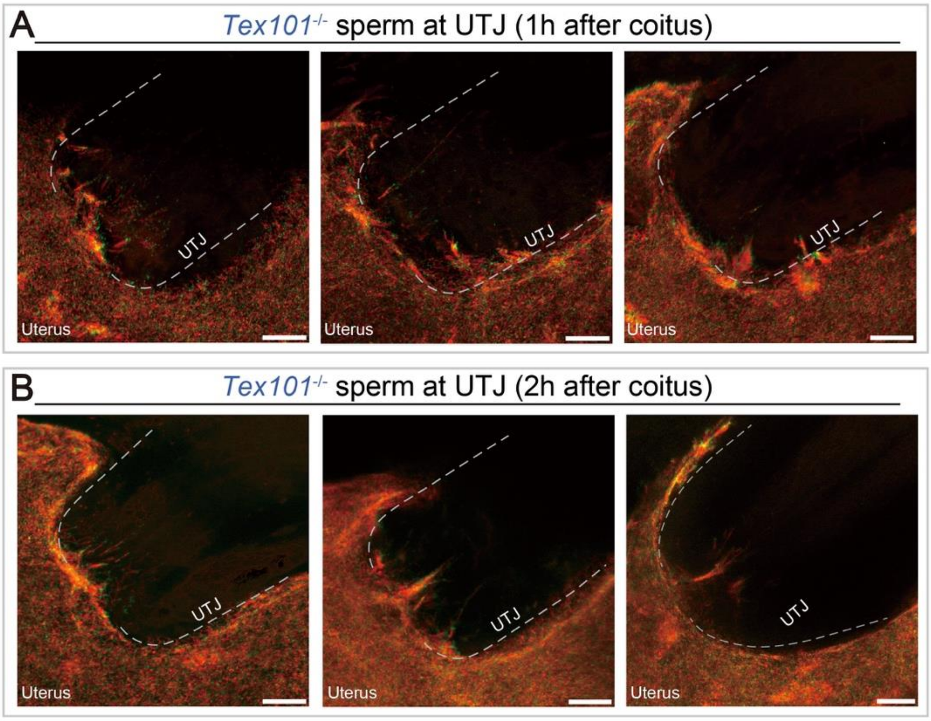

Figure S3. *Tex101$^{-/-}$* sperm behavior at UTJ. (A) *Tex101$^{-/-}$* sperm behavior at UTJ 1h after coitus. (B) *Tex101$^{-/-}$* sperm behavior at UTJ 2h after coitus. Scale bars, 200μm.

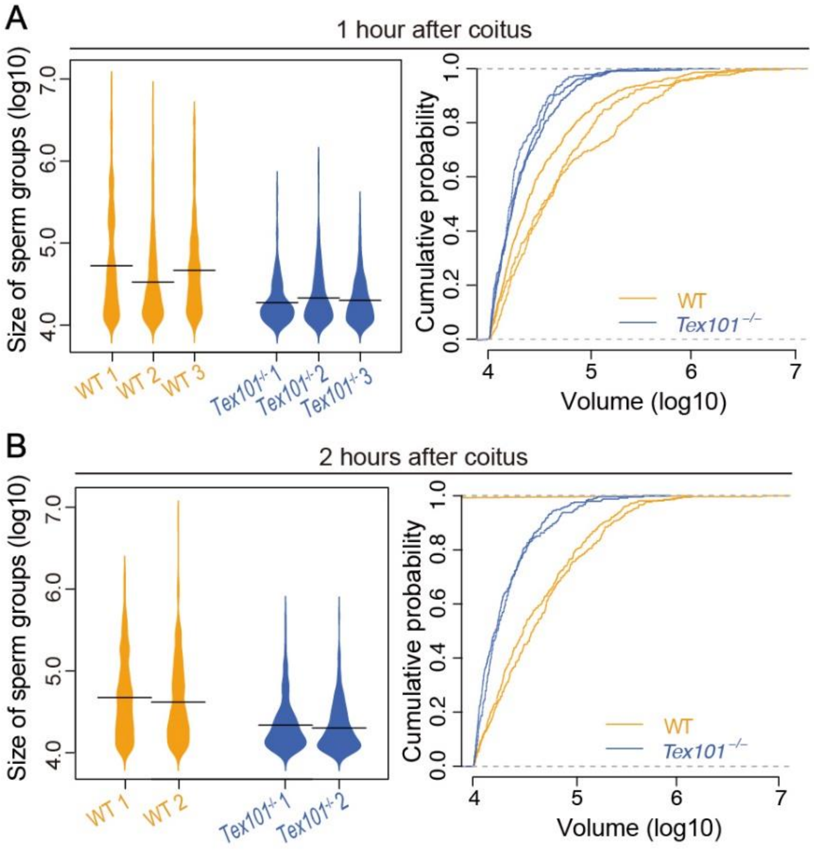

Figure S4. Quantitative comparison of the size of sperm groups (volumetric analysis of the sperm fluorescent signal) in uterus. WT sperm aggregated into sperm clusters and scattered Tex101-/- sperm at one (three WT and three Tex101-/- replicates) (A) and two hours (two WT and two Tex101-/- replicates) (B) after coitus, respectively. Left panel is a violin plot and the right panel is the cumulative distribution. Results are shown as the mean ± SEM , n=3 male mice in (A), n=2 male mice in (B).

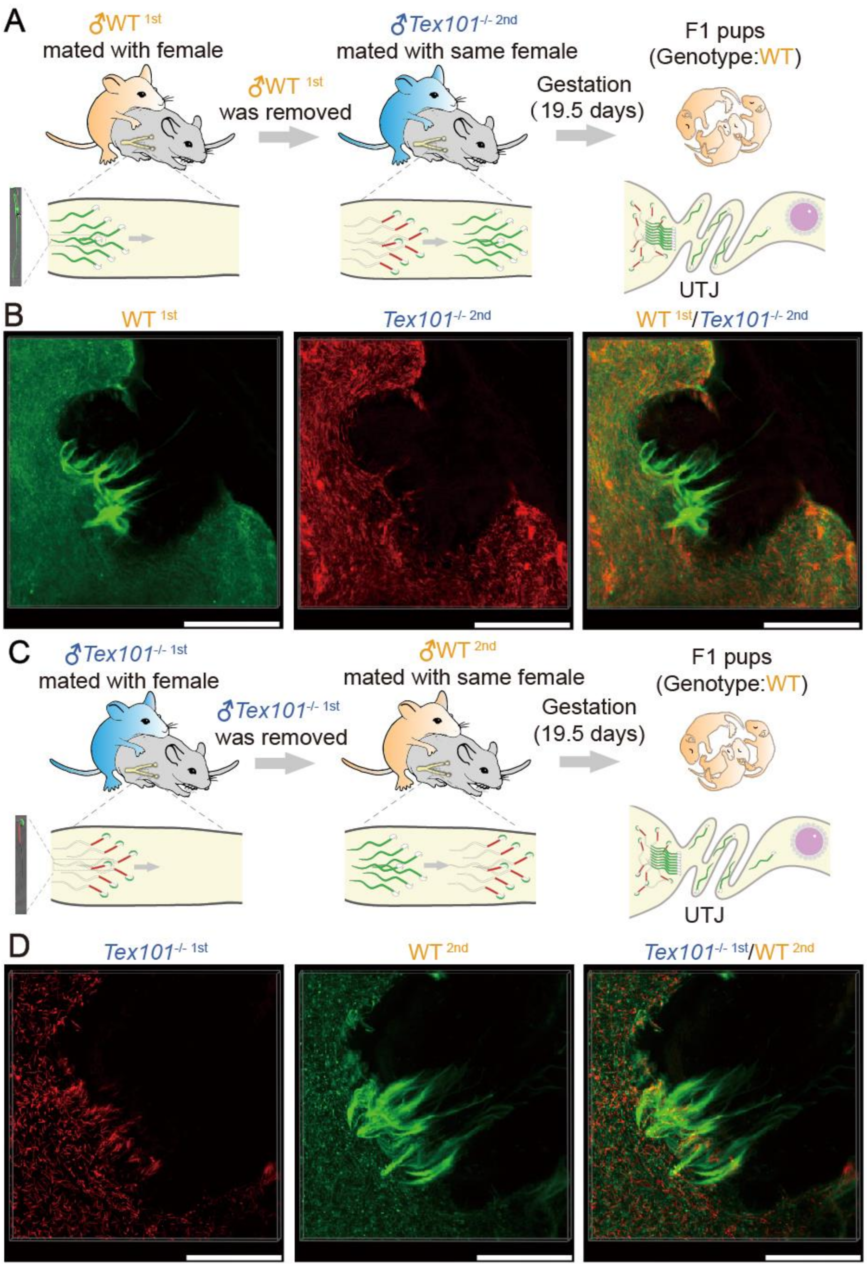

Figure S5. Sequential mating experiments showing that sperm clustering is necessary for passing through the UTJ. (A, C) Schematic diagrams of sequential mating experiment. In (A) the female mated first with the WT male and then with the Tex101-/- male (WT1st, Tex101-/-2nd). In (C) the female mated first with the Tex101-/- male and then with the WT male (Tex101-/-1st, WT2nd). (B, D) 3D visualization of fluorescently labeled WT and Tex101-/- sperm behavior at the UTJ after sequential mating. In (B) the female mated first with the WT male and then with the Tex101-/- male (WT1st, Tex101-/-2nd). In (D) the female mated first with the Tex101-/- male and then with the WT male (Tex101-/-1st, WT2nd). Scale bars, 200μm.
